# Effects of the Characteristics and Duration of Chronic Pain on Psychosomatic Function in the Community-Dwelling Elderly Population

**DOI:** 10.1155/2020/4714527

**Published:** 2020-04-07

**Authors:** Mitsumasa Hida, Misa Nakamura, Masakazu Imaoka, Hidetoshi Nakao, Fumie Tasaki, Tomoko Omizu, Masatoshi Takeda, Tadasuke Ohnishi, Chikamune Wada

**Affiliations:** ^1^Department of Rehabilitation, Osaka Kawasaki Rehabilitation University, Mizuma 158, Kaizuka, Osaka 597-0104, Japan; ^2^Cognitive Reserve Research Center, Osaka Kawasaki Rehabilitation University, Mizuma 158, Kaizuka, Osaka 597-0104, Japan; ^3^Graduate School of Life Science and Systems Engineering, Kyushu Institute of Technology, 2–4 Hibikino, Wakamatsu-ku, Kitakyushu 808-0196, Japan; ^4^Frontier Community Rehabilitation Center, Showa-Inan General Hospital, Akaho 3230, Komagane, Nagano 399-4117, Japan

## Abstract

Catastrophic thinking is related to pain intensity and the degree of disability and influences pain care significantly. However, only few studies have investigated the impact of catastrophic thinking on chronic pain (CP) in the community-dwelling elderly population. This study aimed to evaluate the characteristics of CP in the community-dwelling elderly population and to investigate the effects of different periods of CP on cognitive and psychological functions. A total of 187 community-dwelling elderly people met the inclusion criteria and were included in this cross-sectional study. The survey items included demographic data (age and gender), pain-related questionnaires, psychological and cognitive functions, and sleep status. The duration of CP was investigated using three categories: no pain and pain for ≤1 year and ≥1 year. A logistic regression analysis was performed to identify the factors most strongly associated with the presence of CP. The difference in each assessment was compared according to duration of CP among the three groups and analyzed using the chi-square test, Kruskal–Wallis test, and one-way analysis of variance. The PCS scores and depression scores were significantly higher in long duration of CP compared with no pain and pain for ≤1 year. The present study is consistent with the fear-avoidance model and was concluded that community-dwelling elderly people with CP are depressive and tend to magnify their pain with long duration of CP.

## 1. Introduction

Chronic pain (CP), defined as “pain that extends beyond the expected period of healing or progressive pain due to noncancer diseases,” generally lasts for more than 12 weeks [1, 2]. It is reported to be affected by genetic, environmental, and cultural factors, in addition to socioeconomic status and psychological factors [3]. This suggests that CP is caused by a variety of experiences that affect the individual, regardless of age.

CP is present in 20%–25% of the total population and is related to decline in the quality of life (QOL) and physical functions [4]. Nakamura et al. revealed that CP is associated with a decrease in all domains of the 36-Item Short Form Survey (SF-36) questionnaire QOL score [5]. Additionally, 10% of patients with chronic musculoskeletal pain are limited from attending school or work and require long-term treatment, thereby significantly impacting the medical economics [5]. In Japan, rapid aging of the population has increased the number of community-dwelling elderly people who require long-term care due to decline in psychosomatic functions [6, 7]. Previous studies have shown that CP is associated with increased isolation and fatigue and decreased subjective health, ADL, and motor functions in community-dwelling elderly people [8–10].

Cheng et al. revealed that catastrophic thinking mediates the relationship between pain intensity and depressive symptoms and self-efficacy mediates the relationship between pain intensity and catastrophic thinking [11]. Catastrophic thinking of pain was proposed by Ellis [12] and is defined as “an exaggerated negative mental set brought to bear during actual or anticipated painful experience”; it is a typical cognitive factor for CP [13]. It has been indicated that catastrophic thinking is related to the pain intensity and the degree of disability and has an important influence on the care of pain [14, 15]. However, only few studies have investigated the impact of catastrophic thinking on CP in the community-dwelling elderly population [16, 17]. Hirase et al. revealed that the factor most strongly associated with musculoskeletal CP in community-dwelling elderly people who exercise regularly is the helplessness subscale of the Pain Catastrophizing Scale (PCS) [16]. However, there are individuals who do not exercise regularly in the community-dwelling elderly people; therefore, it is necessary to investigate the general community-dwelling elderly people. Breivik et al. conducted a survey comprising 4839 subjects with CP in Europe; a total of 40% of those with CP did not visit the hospital and only 2% were treated by pain management specialists. One-third of them revealed that they were not currently receiving treatment [18]. In Japan, reports suggest that only 42% of patients with CP receive folk remedies and treatment at medical facilities [5]. The community-dwelling elderly who remain under sufficient care may have negative health-economic impacts as they progress to CP and visit the healthcare facility only after the symptoms worsen. Investigating the cognitive and psychological characteristics, including catastrophic thinking, in individuals with long CP is important for the planning of appropriate prevention and early intervention. Therefore, this study aims to evaluate the characteristics of CP in the community-dwelling elderly and to investigate the effects of different periods of CP on cognitive and psychological functions.

## 2. Materials and Methods

### 2.1. Subjects

This cross-sectional study was approved by the Osaka Kawasaki Rehabilitation University Research Ethics Review Board (approval number: OKRU-A016), and written informed consent was obtained from each participant prior to study inclusion. A total of 215 community-dwelling elderly people who underwent health checks in Kaizuka City, Osaka, from August to September 2018 participated in this study. Participant inclusion criteria were as follows: aged ≥65 years and living at home. Individuals unable to respond to interview questions because of a cognitive impairment were excluded. Of the 215 potential participants, 21 (9.8%) were excluded because their age was under 65 years and 7 (3.3%) were excluded because the data provided were incomplete; therefore, 187 participants (87.0%) met the inclusion criteria and were included in the study (average age, 74.7 ± 6.0 years; males, 47; females, 140). The presence or absence of CP was ascertained in accordance with the following definition of CP: associated with a lesion that persists or recurs beyond 3 months or persists beyond 1 month after recovery from acute tissue damage or does not cure [2]. The subjects were required to indicate the presence or absence of pain by responding to “Yes” and “No” questions in a self-reported questionnaire; those who answered “Yes” were enrolled in the study.

### 2.2. Methods

The survey items included in this study were demographic data (age and gender), pain-related surveys, psychological functions, cognitive functions, and sleep status. The duration of CP was investigated using three categories: no pain, pain for less than 1 year, and pain for more than 1 year. The PCS, a 13-item self-reported measure comprising rumination, magnification, and helplessness, was used in this study [13]. The psychological function assessment was based on the Geriatric Depression Scale 15 (GDS-15) [19]. It is a test consisting of 15 items with “Yes” or “No” answers. Each item is calculated as 1 point with 15 points in total; the higher the score, the more severe the depression. Cognitive functions were investigated using the Mini-Mental State Examination, which consisted of items such as orientation of time and place, memorizing ability, calculation, recall ability, sentence creation, and graphic replication [20]. It is evaluated using a 30-point scale wherein the lower the score, the lower the cognitive function. The sleep state was investigated using the Japanese version of the Pittsburgh Sleep Quality Index (PSQI-J), a questionnaire composed of 18 items about sleep quality, which was developed by Buysse et al. [21, 22]. The questionnaire consists of questions about sleep habits and sleep quality over the past month with a cutoff value of six points. The PSQI is reported to have good validity and to be highly reliable. Additionally, the number of falls over the past year was investigated.

### 2.3. Statistical Analysis

The Shapiro–Wilk test was used to confirm the normality of the distribution of each evaluation item. The endpoints in the CP and non-CP (NCP) groups were compared using the chi-square test, Mann–Whitney's *U* test, and unpaired *t*-test. A logistic regression analysis was performed to identify the factors most strongly associated with the presence of CP. Variance inflation factors for independent variables were calculated to assess multicollinearity between the independent variables. A variance inflation factor >10 was considered to show multicollinearity. Comparison of about the difference in each evaluation item according to duration of CP was compared among the three groups and analyzed using the chi-square test, Kruskal–Wallis test with Scheffe comparison, and one-way analysis of variance with Bonferroni comparison. The statistical software used was IBM SPSS Statistics 26 (IBM Corp., Armonk, NY, US) with a significance level of less than 5%.

## 3. Results

Table 1 shows the results of the univariate analysis in the CP and NCP groups. The total PCS score, rumination, helplessness, magnification, GDS, and number of falls were significantly different between the two groups. The results of the logistic regression analysis are shown in Table 2. Analyses were conducted with CP presence as the dependent variable. Items demonstrating significant differences in previous between-group comparisons were designated as the independent variables. Variance inflation factors for all independent variables were <4, indicating no multicollinearity between the variables. Based on this analysis, PCS rumination score (odds ratio = 1.11) and number of falls (odds ratio = 1.37) were identified as significant factors associated with the presence of CP.

The NCP group comprised 102 patients, the group with pain for less than 1 year comprised 27 patients, and the group with pain for more than 1 year consisted of 58 patients (Table 3). The items that appeared to exert the main effects were total PCS score, rumination, helplessness, magnification, and PSQI-J. The total PCS scores, magnification, and helplessness in the two groups with CP (less than and more than 1 year) were significantly different from those in the NCP group. In addition, GDS was significantly higher in the group with CP for more than 1 year compared with the NCP group. Furthermore, in the group with CP for ≥1 year, GDS and PCS magnification was significantly higher than that in in the group with CP for ≤1 year.

## 4. Discussion

Vlaeyen and Linton advocated the fear-avoidance model as a vicious circle of pain according to which pain causes catastrophe by negatively impacting the thoughts, emotions, cognition, and behavior; this gradually leads to the development of CP as a result of inactivity, depression, and reduced ability, thereby worsening the symptoms [23]. The results of the present study support the fear-avoidance model. Individuals with CP had higher PCS scores than those with NCP, leading to progressive catastrophic pain. Furthermore, the scales for evaluating depressive symptoms and number of falls were also significantly higher among those with CP. PCS rumination was found to be related to CP in the elderly living in the region. Rumination refers to the tendency to repeatedly think about the pain [24]. Previous studies suggest that the subitems of PCS are strongly associated with pain intensity and interference [25, 26]. Pain interference refers to the extent to which pain limits or interferes with an individual's physical, mental, and social activities. Our results support the study by Adachi et al., wherein Japanese individuals tended to concentrate on pain sensation, thus, having a negative impact on daily life [26].

The vicious circle of the fear-avoidance model is presumed to be accompanied by a certain period. Therefore, the disability could be worsened when the duration of CP is prolonged. The findings of the current study indicate that early intervention is required for CP in the community-dwelling elderly. First, all the subscales of the PCS were higher in patients with CP compared with those in the NCP group; the scores were higher as the duration of CP increased. Second, GDS, which evaluates depressive symptoms, was significantly higher in the group with CP for over 1 year compared with the group with NCP. It is well known that catastrophic thinking has a significant effect on depression, stress, functional ability, and disability [27, 28]. In addition, the results of this study revealed that all of the PCS subscale scores were higher and the depression scores were significantly higher when CP persisted for more than one year. Therefore, community-dwelling elderly people with CP must receive appropriate care within a year. The group with CP for more than 1 year had significantly higher PCS magnification and GDS scores than those with CP for less than 1 year. Magnification refers to the tendency to magnify the intensity of pain and the problems caused by it [4]. Rodero et al. investigated the relationship between catastrophic thinking and pain duration in patients with <2 years, 2–4 years, and >4 years of fibromyalgia and found that rumination was a significant predictor in the <2 year group, magnification and helplessness in the period of 2–4 years group, and helplessness in the >4 years group [29]. In other words, differences in the duration of CP can affect the subscale of catastrophic thinking. Our findings did not agree with those reported by Rodero et al. because the subjects in the current study comprised community-dwelling elderly people. However, initially our study population did present with significantly higher values for all the PCS items. When the duration of pain exceeded 1 year, the number of magnified items and the intensity of pain increased, suggesting the presence of mental disorders such as depression.

The limitation of this study is that it has not investigated the characteristics of subjects with CP for more than a year. It has been reported that the morbidity of knee osteoarthritis, lumbar spondylosis, and osteoporosis, which are cited as one of the causes of CP, increases with age [30]. Therefore, the subjects of this study who were assigned to groups with CP of more than 1 year may have experienced CP for a longer period. In addition, subjects who have CP for a longer period may not have been able to participate in this study due to a decline in mobility. Additional surveys including these patients need to be conducted.

In this study, we found that Japanese patients with CP have a high degree of resistance to pain, and as the period of CP increases, the tendency to magnify the pain increases. This exerts an adverse effect on daily activities and social functions. Rehabilitation with activities such as physical activity, exercise therapy, and cognitive behavior therapy is important for the treatment of CP [31]. These can be implemented by local residents; hence, it is important to raise the awareness of health workers in the community.

## 5. Conclusions

We evaluated the characteristics of CP in the community-dwelling elderly and investigated the effects of different periods of CP on cognitive and psychological functions. PCS rumination score and number of falls were identified as significant factors associated with the presence of CP. Additionally, individuals with longer CP were suggested to have a worse PCS rumination score and GDS. Early care must be provided to community-dwelling elderly population with CP.

## Figures and Tables

**Table 1:** Comparison between groups based on chronic pain.

			NCP	CP	Total
Subject			102	85	187
Age (year)			74.7 ± 6.0	74.4 ± 5.9	74.7 ± 6.0
Sex		Male	28 (27.5%)	19 (22.4%)	47 (25.1%)
PCS (points)		Rumination	5.2 ± 5.8	10.4 ± 5.9^*∗∗*^	7.6 ± 6.4
		Magnification	1.9 ± 2.8	4.2 ± 3.5^*∗∗*^	3.0 ± 3.3
		Helplessness	2.6 ± 3.4	6.0 ± 5.2^*∗∗*^	4.1 ± 4.6
		Total score	9.7 ± 10.9	20.5 ± 13.4^*∗∗*^	14.5 ± 13.2
GDS (points)			2.8 ± 2.2	3.8 ± 2.8^*∗*^	3.3 ± 2.5
Number of falls (frequency)			0.3 ± 0.8	1.2 ± 3.1^*∗∗*^	0.7 ± 2.2
PSQI (points)			5.1 ± 3.2	5.7 ± 4.0	5.4 ± 3.6
MMSE (points)			27.9 ± 2.8	28.2 ± 2.3	28.0 ± 2.6

Mean ± SD, ^*∗*^*p* < 0.05, and ^*∗∗*^*p* < 0.01. CP: chronic pain, NCP: nonchronic pain, PCS: Pain Catastrophizing Scale, GDS: Geriatric Depression Scale, PSQI: Pittsburgh Sleep Quality Index, and MMSE: Mini-Mental State Examination.

**Table 2:** Multiple logistic regression analysis for odds ratio and 95% confidence interval of chronic pain.

		Odds ratio	95% confidential interval	*P* value
PCS	Rumination	1.11	1.01–1.21	0.02
	Magnification	0.97	0.80–1.16	0.97
	Helplessness	1.10	0.97–1.24	0.14
Number of falls		1.37	1.01–1.86	0.04
GDS		1.07	0.92–1.23	0.38

Data were adjusted by age and sex. PCS: Pain Catastrophizing Scale and GDS: Geriatric Depression Scale.

**Table 3:** Comparisons between nonchronic pain, chronic pain (less than 1 year), and chronic pain (more than 1 year) groups.

		NCP (*N* = 102)	CP		NCP vs. CP (≤1 year)	NCP vs. CP (≥1 year)	CP (≤1 year) vs. CP (≥1 year)
			≤1 year (*N* = 27)	≥1 year (*N* = 58)			
Age^*a*^		74.7 ± 6.0	73.8 ± 5.8	75.1 ± 6.0	Ns	Ns	Ns
Sex^*c*^	Male	28 (27.5%)	4 (14.8%)	15 (25.9%)	Ns	Ns	Ns
PCS^*b*^	Rumination	5.2 ± 5.8	8.9 ± 5.6	11.1 ± 5.9	<0.01	<0.01	Ns
	Magnification	1.9 ± 2.8	2.6 ± 2.8	4.9 ± 3.6	Ns	<0.01	<0.01
	Helplessness	2.6 ± 3.4	5.0 ± 5.0	6.4 ± 5.3	<0.05	<0.01	Ns
	Total	9.7 ± 10.9	16.4 ± 12.0	22.4 ± 13.7	<0.05	<0.01	Ns
GDS^*b*^		2.8 ± 2.2	2.7 ± 1.7	4.3 ± 3.0	Ns	<0.05	<0.01
Number of falls^*b*^		0.3 ± 0.8	0.5 ± 0.9	1.5 ± 3.7	Ns	<0.01	Ns
PSQI^*b*^		5.1 ± 3.2	5.0 ± 4.0	6.1 ± 4.0	Ns	Ns	Ns
MMSE^*b*^		27.9 ± 2.8	28.1 ± 2.7	28.3 ± 2.1	Ns	Ns	Ns

^*a*^One-way ANOVA (multiple comparison: Bonferroni); ^*b*^Kruskal–Wallis test (multiple comparison: Scheffe); ^*c*^chi-square test. CP, chronic pain; NCP, nonchronic pain; PCS, Pain Catastrophizing Scale; GDS, Geriatric Depression Scale; PSQI, Pittsburgh Sleep Quality Index; MMSE, Mini-Mental State Examination.

## Data Availability

The data used to support the findings of this study are available from the corresponding author upon request.
